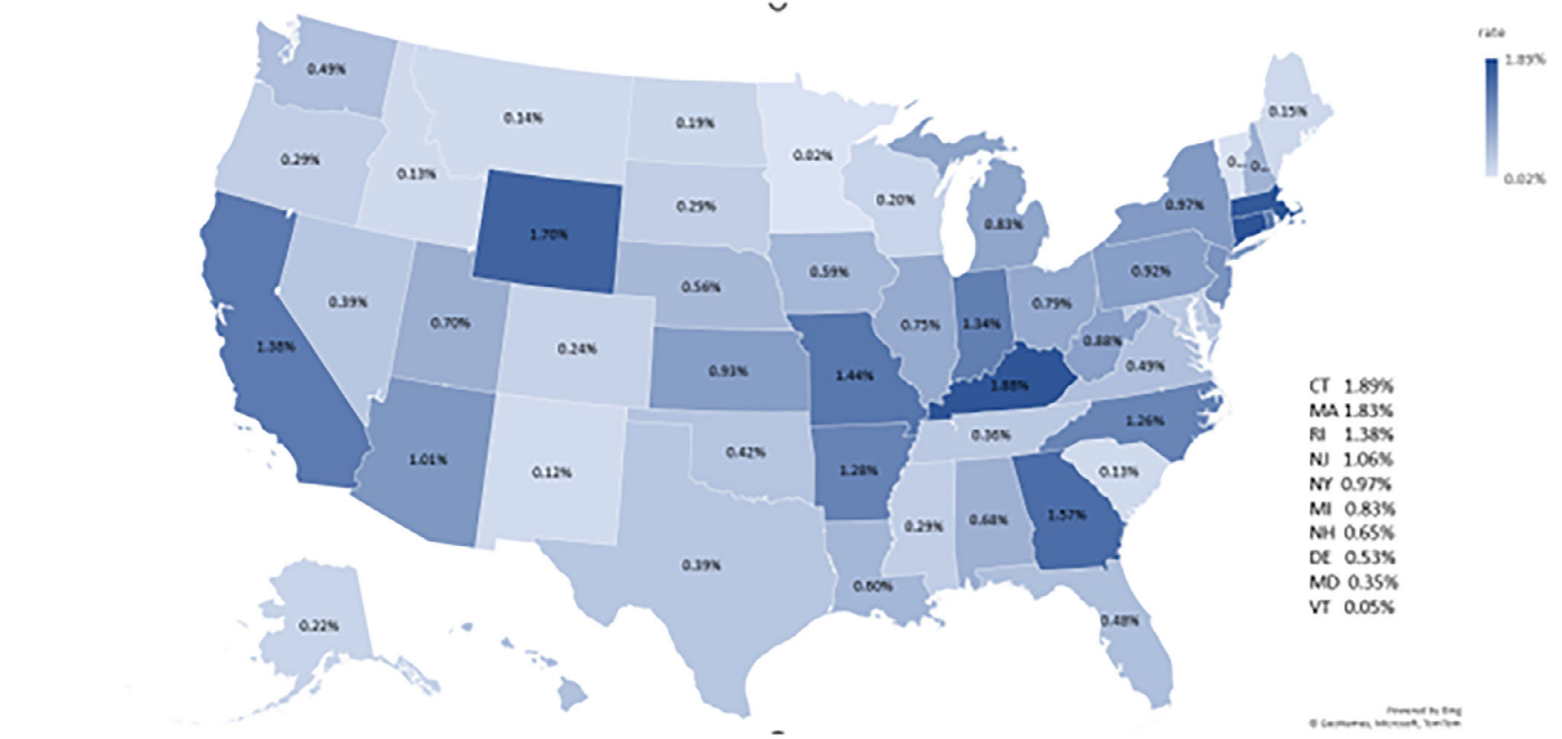# Lecanemab adoption and geographic variation among patients with diagnosis of mild cognitive impairment or Alzheimer's dementia in Medicare fee‐for‐service beneficiaries

**DOI:** 10.1002/alz70860_106870

**Published:** 2025-12-23

**Authors:** Amir Abbas Tahami Monfared, Raymond Zhang, Quanwu Zhang, Ran Gao, Babak Haji

**Affiliations:** ^1^ Alzheimer's Disease & Brain Health, Eisai Inc., Nutley, NJ, USA; ^2^ Clinical Evidence Generation, Deep Human Biology Learning, Eisai Inc., Nutley, NJ, USA; ^3^ Alzheimer's Disease Franchise, Eisai Inc., Nutley, NJ, USA; ^4^ Eisai, Nutley, NJ, USA

## Abstract

**Background:**

Lecanemab for treating early Alzheimer's disease has been available in the United States since January 2023. This study aimed to understand patient characteristics and geographic variation among US Medicare fee‐for‐service beneficiaries with an ICD‐10 diagnosis code for mild cognitive impairment (MCI) or Alzheimer's dementia (AD) who received lecanemab infusion(s) during January ‐ September 2024.

**Method:**

Access to “preview claims” (Jan 1 – Sep 30, 2024) was granted by the Centers for Medicare and Medicaid Services. To determine lecanemab infusion rate, the number of patients with a J0174 HCPCS code were identified and divided by the total number of patients with ≥1 ICD‐10 code for MCI/AD with all disease stages. We described patient demographics and comorbidities between patients with ≥1 lecanemab infusion and those without, and patient residence locations as urban vs rural settings.

**Result:**

Patients (*N* = 2,512) receiving lecanemab infusion had a mean age of 75.4 (standard deviation = 5.7) years as compared to 80.8 (8.9) years in patients with MCI/AD not receiving the infusion. Women consisted of 53.1% the infusion group vs 62.2% in the non‐infusion group. The infusion group had 91.1% White, 1.1% Black, 0.2% Hispanic, 1% Asian compared to 86% White, 6.7% Black, 1.8 Hispanic, 1.6% Asian in the non‐infusion group. Comorbidity rates were generally lower for the infusion vs non‐infusion group. Specially, hypertension was 4.5 vs 63%, hyperlipidemia 3.8 vs 47.8%, diabetes 1.7 vs 24.4%, cerebrovascular disease 1.1 vs 14.7%, and congestive heart failure 0.1 vs 11.6% for infusion vs non‐infusion group. Among infusion group, 88.7 vs 11.3% were in urban vs rural areas. The states having a higher lecanemab infusion rate include Connecticut (1.9%), Kentucky (1.9%), Massachusetts (1.8%), Wyoming (1.7%), Georgia (1.6%), Missouri (1.4%), California (1.4%), Rhode Island (1.4), Indiana (1.3%), and New Jersey (1.1%).

**Conclusion:**

Although lecanemab infusion rate was greater than 1% among only 10 states, the therapy adoption rate among the target population is likely higher as the infusion rate denominator included unconfirmed Alzheimer's cases and patients beyond the early disease stage. Continued medical/scientific education plus availability of home infusion and self‐injectable delivery formulation are expected to support increasing lecanemab adoption, especially among minority groups.